# Diverse Functions of Extracellular Vesicles in Ovarian Cancer

**DOI:** 10.31662/jmaj.2025-0086

**Published:** 2025-06-13

**Authors:** Akira Yokoi

**Affiliations:** 1 Department of Obstetrics and Gynecology, Nagoya University Graduate School of Medicine, Nagoya, Japan; 2 Nagoya University Institute for Advanced Research, Nagoya, Japan; 3 Japan Science and Technology Agency, FOREST, Saitama, Japan

**Keywords:** ovarian cancer, extracellular vesicle, exosome

## Abstract

Extracellular vesicles (EVs) play various roles in cancer progression, mediating intercellular signaling. EVs carry bioactive substances such as ribonucleic acid, deoxyribonucleic acid, and proteins, each providing important functions in whole biological fields, not only in cancer. Particularly in clinical oncology, EVs have attracted much attention owing to their promising potential as new diagnostic and therapeutic targets. Ovarian cancer is one of the major causes of gynecologic cancer deaths and is often asymptomatic in the early stages; therefore, biomarkers that enable early diagnosis are critical to improving patient survival. Ovarian cancer cells easily spread through the abdominal cavity rather than through the bloodstream, forming direct metastatic foci in organs such as peritoneal membranes or omentum. The disease is treated multimodally with surgery and chemotherapy, even in advanced stages, and a certain degree of response to treatment is observed. However, most relapsed cancer gradually acquired resistance to chemotherapies. We have investigated various EV molecules in ovarian cancer and shown their clinical utility. In addition, the heterogeneity of EVs has emerged as a recent topic, and we have developed and used novel techniques to understand them. Applying these findings to clinical practice, EV-based approaches have the potential to revolutionize ovarian cancer management, enabling early detection, personalized monitoring, and targeted therapy. Continued innovation and interdisciplinary collaboration will be key to realizing the full potential of EV translational research to improve outcomes for patients with ovarian cancer.

## Ovarian Cancer

Ovarian cancer is one of the most lethal gynecological malignancies, accounting for more than 150,000 deaths globally each year. High-grade serous ovarian carcinoma (HGSOC), the most aggressive subtype, accounts for approximately 75% of epithelial ovarian cancer cases. A major contributor to its high mortality rate is the lack of early detection methods because the disease often presents with non-specific symptoms such as abdominal discomfort, bloating, and fatigue. Consequently, approximately 70% of ovarian cancer cases are diagnosed at advanced stages (III/IV), when the disease has disseminated throughout the peritoneal cavity. In these cases, survival outcomes remain poor, with a 5-year survival rate of less than 45%. In contrast, early-stage detection (stage I) confers a survival rate higher than 90%, underscoring the critical need for effective biomarkers and diagnostic tools. Ovarian cancer is characterized by unique patterns of dissemination. Unlike other solid tumors, ovarian cancer metastasizes primarily through the peritoneal cavity rather than the bloodstream, facilitated by tumor cells shedding into ascitic fluid. These cells interact with peritoneal mesothelial cells and establish metastatic lesions on organ surfaces, including the pelvic peritoneum, or diaphragm. The molecular mechanisms underlying peritoneal dissemination involve intricate communication between cancer cells and their microenvironment. The tumor microenvironment plays a critical role in cancer metastasis by facilitating interactions between cancer cells and surrounding stromal cells, immune cells, and extracellular matrix components. These interactions help cancer cells acquire invasive properties, escape the primary tumor, and colonize distant organs. In addition, drug resistance, especially in platinum-based agents, is also a major challenge, and one key contributor to drug resistance is cancer-associated fibroblasts. The fibroblasts secrete various factors that activate survival pathways in cancer cells. This activation enhances resistance to chemotherapy and targeted therapies by promoting cell proliferation and inhibiting apoptosis. To overcome refractory ovarian cancer, the new therapeutic modality for understanding cancer malignancy is needed.

## Extracellular Vesicles

**Figure 1.**
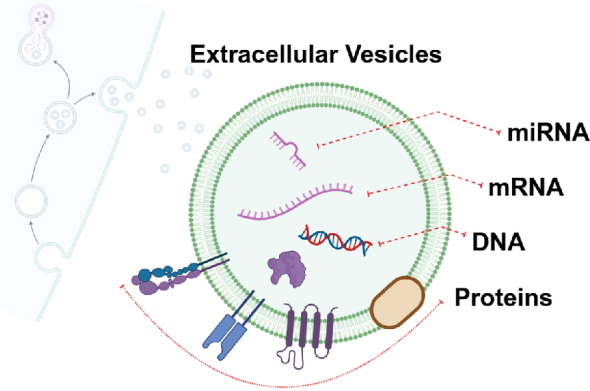
Diverse target molecules on EVs. DNA: deoxyribonucleic acid; EV: extracellular vesicle; mRNA: messenger ribonucleic acid; miRNA: micro ribonucleic acid.

Extracellular vesicles (EVs) are lipid bilayer-bound particles actively released by cells into the extracellular space. They range in size and include exosomes (50-200 nm), microvesicles (200-1,000 nm), and apoptotic bodies (>1,000 nm). EVs act as messengers in intercellular communication by carrying a diverse cargo of proteins, lipids, metabolites, and nucleic acids (ribonucleic acid [RNA] and deoxyribonucleic acid [DNA]) (Figure 1). These molecules are selectively packaged into EVs and delivered to recipient cells, where they influence gene expression, signaling pathways, and cellular behavior. In cancer biology, EVs play a crucial role in disease progression by promoting metastasis, immune modulation, angiogenesis, and resistance to therapy. Specifically, tumor-derived EVs alter the microenvironment to favor tumor growth and dissemination. In ovarian cancer, EVs shed into ascitic fluid interact with mesothelial cells, disrupt the peritoneal barrier, and facilitate the adhesion and invasion of tumor cells. In addition, EVs prepare metastatic niches on distant organ surfaces by reprogramming stromal and immune cells. Given their ability to circulate in bodily fluids, EVs have garnered significant attention as potential biomarkers for non-invasive cancer diagnosis and monitoring. The molecular content of EVs―RNA, DNA, and protein―reflects the physiological and pathological states of their parent cells, enabling them to serve as liquid biopsy tools.

## EV-RNAs

The RNA cargo of EVs, particularly microRNA (miRNA) and messenger RNA (mRNA), plays pivotal roles in ovarian cancer biology. Tumor-derived EVs selectively package RNA molecules that influence recipient cells, driving processes such as proliferation, metastasis, and immune evasion. One significant finding is the role of MMP1 mRNA in peritoneal dissemination. EVs derived from highly metastatic ovarian cancer cells deliver intact MMP1 mRNA to mesothelial cells, inducing apoptosis and disrupting the peritoneal barrier. This facilitates tumor cell adhesion and subsequent peritoneal metastasis, underscoring the functional importance of EV-mRNA in cancer progression ^(1)^.

EV-miRNAs have also emerged as key regulators and biomarkers in ovarian cancer. Our research group explored the potential of serum miRNAs as biomarkers for ovarian cancer detection ^(2)^. Serum miRNA profiles from 4046 samples, including 428 ovarian tumor cases, were analyzed using microarrays. A diagnostic model based on ten miRNAs was developed and validated, achieving exceptional accuracy (sensitivity: 0.99, specificity: 1.00). Two additional models were constructed: one distinguishing ovarian cancer from other malignancies and another differentiating it from benign and borderline ovarian tumors. The first model effectively identified ovarian cancer, but the second faced challenges in discriminating malignant from benign tumors. These findings indicate the feasibility of serum miRNA profiling for ovarian cancer screening. Although highly accurate in detecting cancer, further refinements are needed to improve specificity in distinguishing benign conditions. Large-scale prospective studies will be essential to validate clinical applicability and establish miRNA-based diagnostics as a non-invasive screening tool.

## EV-DNAs

The presence of DNA in EVs, particularly genomic DNA, has significant implications for ovarian cancer biology. EV-DNA reflects the genetic landscape of the parent tumor, including copy number variations (CNVs) and mutations. This makes EV-DNA a valuable tool for tumor monitoring and non-invasive genomic profiling.

Recent studies have revealed that EVs carry nuclear-derived DNA fragments, a process linked to micronuclei (MN) formation ^(3)^. MN are cytoplasmic structures containing fragmented chromosomes, which arise from genomic instability―a hallmark of ovarian cancer. Under genotoxic stress, MN collapse and release nuclear content into the cytoplasm, where it is incorporated into multivesicular bodies and secreted as part of EVs. In ovarian cancer, EV-derived DNA mirrors the CNV profiles of the primary tumor. Whole-genome sequencing of EV-DNA revealed high concordance with tumor DNA, highlighting its potential for detecting genetic alterations and tracking tumor evolution. This approach offers a non-invasive alternative to traditional biopsies, particularly in patients with advanced-stage disease or inaccessible tumors.

## EV Proteins

EV-associated proteins are critical for both biomarker discovery and functional studies in ovarian cancer. Proteomic analyses have identified specific EV proteins that are enriched in tumor-derived EVs and associated with disease progression. Our research group newly identified FRα, claudin-3, and TACSTD2 as HGSOC-specific EV proteins ^(4)^. These proteins are highly expressed in small EVs (sEVs) and can distinguish ovarian cancer-derived EVs from benign or non-cancerous sources. The detection of these proteins highlights their potential as clinical biomarkers for early detection and disease monitoring. In general, EV proteins play essential functional roles in cancer progression. EVs from highly metastatic ovarian cancer cells deliver pro-apoptotic signals, leading tumor cells to adhere, invade, and establish secondary lesions. Proteomic studies have also revealed differences between sEVs and large EVs, emphasizing the need to study EV subpopulations to fully understand their roles in cancer progression.

## New EV Isolation Devices

Technological innovations have addressed major challenges in EV isolation and analysis, enabling precise characterization of EVs in ovarian cancer. Two notable advancements include cellulose nanofiber (CNF) sheets ^(5)^ and polyketone-coated nanowires (pNWs) ^(4)^. CNF sheets, with tailored porous nanostructures, allow efficient EV capture from trace biofluid volumes and organ surfaces. These sheets enable spatial profiling of EVs, revealing location-specific heterogeneity. For instance, EVs captured from the pelvic peritoneum, liver surface, and tumor surfaces exhibit distinct miRNA profiles, providing insights into tumor progression and metastatic potential. pNWs offer a complementary approach for isolating sEVs from complex biofluids such as serum and ascites. pNWs enable rapid EV purification and multiplexed protein analysis, facilitating the detection of HGSOC-specific EV proteins such as FRα and claudin-3. These technologies represent a significant step forward in EV research, overcoming limitations of traditional isolation methods and enabling the discovery of novel biomarkers and therapeutic targets.

## Future Perspective

The studies reviewed here highlight the critical roles of EVs in ovarian cancer progression, biomarker discovery, and therapy. Moving forward, integrating EV-based analyses into clinical practice will require large-scale validation of EV-derived biomarkers. Technologies such as CNF sheets and pNWs hold immense potential for non-invasive diagnostics, real-time disease monitoring, and spatial EV analysis. Future research should focus on unraveling the functional roles of EV cargo, particularly RNA, DNA, and protein, in tumor progression and metastasis. Moreover, engineering EVs for therapeutic applications, such as targeted drug delivery or immune modulation, represents a promising avenue for translational research. The potential clinical impact of EVs remains in its prologue.

## Article Information

This article is based on the study, which received the Medical Research Encouragement Prize of The Japan Medical Association in 2024.

### Conflicts of Interest

None
